# Supplementary figures and images for: The Anti-Diabetic Drug Metformin Reduces BACE1 Protein Level by Interfering with the MID1 Complex
Source: PLoS One. 2014 Jul 15;9(7):e102420. doi: 10.1371/journal.pone.0102420 (PMC4099345; doi:10.1371/journal.pone.0102420)

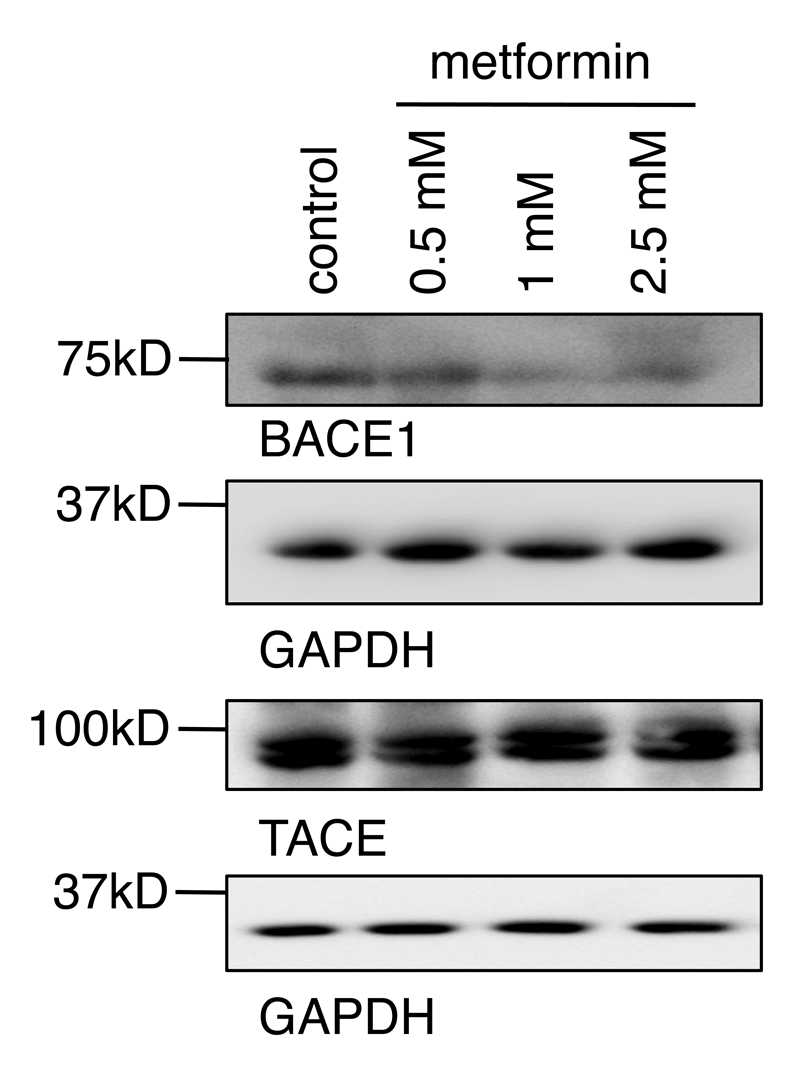

Supplement: Figure S1 — Metformin decreases BACE1 protein level. SH-SY5Y-APPswe cells were treated with increasing concentrations of metformin for 24 h and protein levels were analyzed on western blots using BACE1-, TACE- or β-actin-specific antibodies. (TIF) [file pone.0102420.s001.tif]

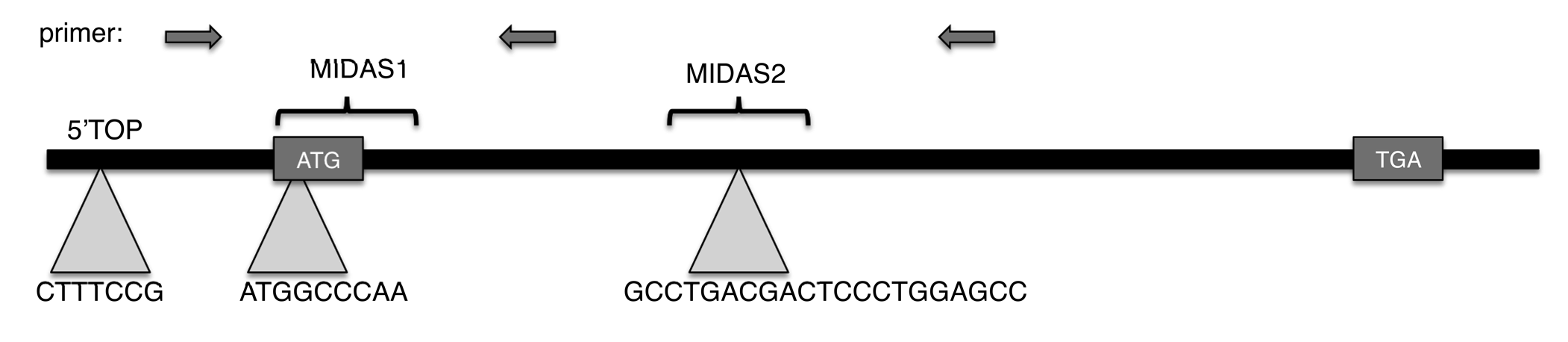

Supplement: Figure S2 — Schematic representation of the BACE1 mRNA transcript. The translational start (ATG) and stop codons (TGA) are indicated. Furthermore the predicted 5′TOP and two of the predicted MIDAS motifs are illustrated. Primers for in vitro amplification and in vitro transcription for mRNA pull-down assays are indicated as arrows. (TIF) [file pone.0102420.s002.tif]

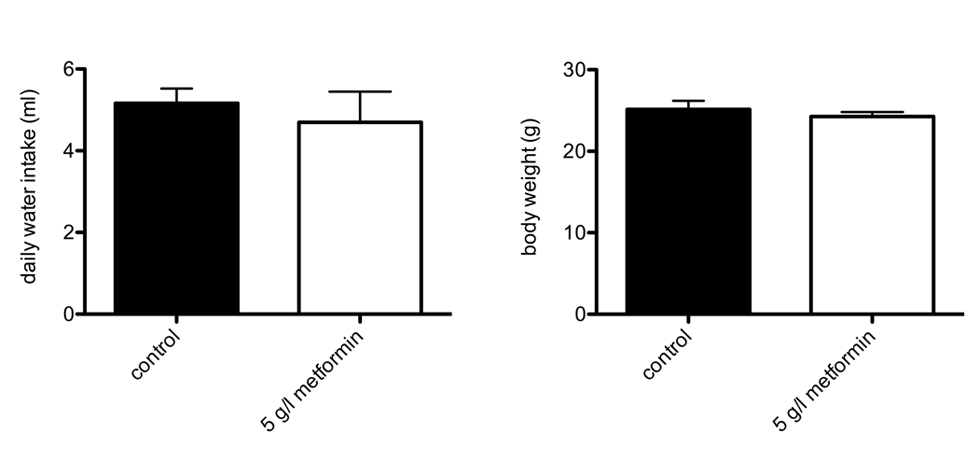

Supplement: Figure S3 — Metformin does not influence body weight or water intake. Wild type mice were treated for 2 weeks with metformin in the drinking water. Body weight and water intake of these experimental animals are shown. Columns represent mean values +/− SEM, (n = 5 per group). (TIF) [file pone.0102420.s003.tif]
