# Supplementary material for: The Anti-Diabetic Drug Metformin Reduces BACE1 Protein Level by Interfering with the MID1 Complex
Source: PLoS One. 2014 Jul 15;9(7):e102420. doi: 10.1371/journal.pone.0102420 (PMC4099345; doi:10.1371/journal.pone.0102420)
Supplement: Table S1 — Primer sequences. Sequences of primers used in this study are listed (in 5′→3′ direction). (DOCX) [file pone.0102420.s004.docx]

| **Primer** | **Gene amplified** | **Sequence** |
| --- | --- | --- |
| mBACE1-for | BACE1 mouse | TCCGGCTCAGAACTACAGTGTAAT |
| mBACE1-rev | BACE1 mouse | TGCGGCGTTTTCATGGT |
| mTACE/ADAM17 for | TACE/ADAM17 mouse | GGCTGGACCCCGTTCCT |
| mTACE/ADAM17 rev | TACE/ADAM17 mouse | TCAGAGGGTACAGAAAAACAGACTGA |
| GAPDH mouse for | GAPDH mouse | GCACAGTCAAGGCCGAGAAT |
| GAPDH mouse rev | GAPDH mouse | GCCTTCTCCATGGTGGTGAA |
| BACE1-T7-for1 | BACE1 human | CCAAGCTTCTAATACGACTCACTATAGGGAGACCCAAGCTCCCTCTCCTGAG |
| BACE1-T7-for2 | BACE1 human | CCAAGCTTCTAATACGACTCACTATAGGGAGATCCAACTGGGAAGGCATCC |
| BACE1-rev | BACE1 human | GAGGCTGCCTTGATGGATTTG |
