# Supplementary material for: Correction: The Anti-Diabetic Drug Metformin Reduces BACE1 Protein Level by Interfering with the MID1 Complex
Source: PLoS One. 2014 Aug 25;9(8):e107080. doi: 10.1371/journal.pone.0107080 (PMC4143310; doi:10.1371/journal.pone.0107080)
Supplement: File S1 — Originally published, uncorrected article. (PDF) [file pone.0107080.s001.pdf]

PLOS ONE

The Anti-Diabetic Drug Metformin Reduces BACE1 Protein Level by Interfering with the MID1 Complex

Moritz M. Hettich[1], Frank Matthes[2], Devon P. Ryan[1], Nadine Griesche[2], Susanne Schröder[1], Stephanie Dorn[2], Sybille Kraußß[2]*, Dan Ehninger[1]*

1 Deutsches Zentrum für Neurodegenerative Erkrankungen e.V. (DZNE), Bonn, Germany, 2 Deutsches Zentrum für Neurodegenerative Erkrankungen e.V. (DZNE), Bonn, Germany

Abstract

Alzheimer's disease (AD), the most common form of dementia in the elderly, is characterized by two neuropathological hallmarks: senile plaques, which are composed of Aβ peptides, and neurofibrillary tangles, which are composed of hyperphosphorylated TAU protein. Diabetic patients with dysregulated insulin signalling are at increased risk of developing AD. Further, several animal models of diabetes show increased Aβ expression and hyperphosphorylated tau. As we have shown recently, the anti-diabetic drug metformin is capable of dephosphorylating tau at AD-relevant phospho-sites. Here, we investigated the effect of metformin on the main amyloidogenic enzyme BACE1 and, thus, on the production of Aβ peptides, the second pathological hallmark of AD. We find similar results in cultures of primary neurons, a human cell line model of AD and *in vivo* in mice. We show that treatment with metformin decreases BACE1 protein expression by interfering with an mRNA-protein complex that contains the ubiquitin ligase MID1, thereby reducing BACE1 activity. Together with our previous findings these results indicate that metformin may target both pathological hallmarks of AD and may be of therapeutic value for treating and/or preventing AD.

Citation: Hettich MM, Matthes F, Ryan DP, Griesche N, Schröder S, et al. (2014) The Anti-Diabetic Drug Metformin Reduces BACE1 Protein Level by Interfering with the MID1 Complex. PLoS ONE 9(7): e102420. doi:10.1371/journal.pone.0102420

Editor: Hemant K. Paudel, McGill University Department of Neurology and Neurosurgery, Canada

Received February 21, 2014; **Accepted** June 18, 2014; **Published** July 15, 2014

Copyright: © 2014 Hettich et al. This is an open-access article distributed under the terms of the Creative Commons Attribution License, which permits unrestricted use, distribution, and reproduction in any medium, provided the original author and source are credited.

Funding: This work was supported by the Else Kröner-Fresenius-Stiftung (2012_A304). The funders had no role in study design, data collection and analysis, decision to publish, or preparation of the manuscript.

Competing Interests: The authors have declared that no competing interests exist.

* Email: dan.ehninger@dzne.de (DE); sybille.krauss@dzne.de (SK)

Introduction

Alzheimer's disease (AD) is characterized by progressive neuronal loss in brain areas underlying memory and higher mental functions and is the most common form of dementia in the elderly. The disease was first described in 1906 by Alois Alzheimer [1,2], who observed two pathological hallmarks in patients' brains: senile plaques, which consist of Aβ peptides, and neurofibrillary tangles, which are composed of hyperphosphorylated TAU protein. Aβ peptides are formed by sequential proteolytic cleavage of the amyloid precursor protein (APP). While the non-amyloidogenic pathway involves sequential cleavage of full-length APP by the α-secretases (TACE, ADAM10) followed by cleavage by the γ-secretase, the first step of the amyloidogenic pathway and therefore in Aβ formation is cleavage of full-length APP by the β-secretase BACE1. The resulting C-terminal fragments (β-CTF) is cleaved by the γ-secretase, which generates Aβ [3]. Multiple lines of evidence have shown that overproduction of Aβ results in neuronal dysfunction and, eventually, neuronal death [4]. BACE1 can cleave APP at two different sites: at Aβ Asp1, producing Aβ in the amyloidogenic pathway; or Aβ Glu11, producing truncated non-amyloidogenic Aβ. Under physiological conditions, BACE1 cleaves APP at Glu11, whereas cleavage at the Asp1-site is enhanced in AD models [2]. BACE1 has a paralogue, BACE2, that shares 64% amino acid similarity. BACE2 cleaves APP at a different site than BACE1, thereby abolishing Aβ production [7]. Furthermore, BACE2 mRNA is expressed at undetectable or very low levels in the human brain [5]. Therefore, while BACE1 is thought to be responsible for neuronal substrate processing, BACE2 serves a similar role in other tissues, such as the pancreas [6]. Together, these findings indicate that BACE2 is of limited relevance in driving AD pathology.

The second pathological hallmark of AD - neurofibrillary tangles - are mainly composed of hyperphosphorylated TAU. Normally, TAU is a microtubule-associated protein that stimulates and stabilizes microtubule assembly. Tau in AD patients' brains shows three- to four-fold higher phosphorylation levels compared to non-AD brains. Upon phosphorylation, TAU dissociates from microtubules, resulting in microtubule destabilization [8,9]. The main TAU phosphatase is protein phosphatase 2A (PP2A), which is capable of dephosphorylating TAU at AD-relevant phospho-sites [10].

Patients who suffer from type 2 diabetes mellitus (DM) are at increased risk of developing AD [11–13] and AD patients show decreased insulin-IGF-1R signalling. The underlying cellular mechanisms for this association are still unclear but insulin signalling resistance is also a typical hallmark of DM. Insulin plays an important role in controlling glucose metabolism. It activates the insulin receptor, which leads to activation of the IRS family of proteins. Phosphorylated IRS then activates its downstream targets. Among them, PI3K has a major role in insulin function, mainly via activation of Akt. Activated Akt induces glycogen synthesis via inhibition of GSK-3, and it induces protein synthesis through mTOR signalling (reviewed in [14–17]). Numerous

animal models for DM have been developed and it has been shown that both Aβ and hyperphosphorylated tau are increased in those DM models [18,19]. Various treatments for DM have been developed and one of the most commonly used oral hypoglycaemic medications is metformin [20,21].

As we have shown recently, metformin is capable of dephosphorylating tau at AD-relevant phospho-sites [22]. Metformin can induce PP2A activity by interfering with the assembly of the MID1-PP2A-complex. MID1 is an E3 ubiquitin ligase that binds to PP2Ac via its regulatory subunit α4 and catalyses the ubiquitin-dependent degradation of PP2Ac [23]. Furthermore, the MID1 protein complex stimulates translation of mRNAs that are associated with this protein complex [24,25]. Upon metformin treatment, the ubiquitin-dependent degradation of PP2Ac is inhibited, resulting in increased PP2A activity and dephosphorylation of tau at AD specific sites [22].

Based on this observation, we wanted to analyze the effects of metformin on the second pathological hallmark of AD, the senile plaques, which are composed of Aβ peptides. Here we show that metformin decreases BACE1 protein levels in both cell culture models and *in vivo*. Metformin dephosphorylates the translation regulator S6, thereby reducing BACE1 protein translation. As a result of this decrease in BACE1 protein level, the cleavage of APP is reduced. Our data suggest a potentially beneficial role of metformin in the prophylaxis or therapy of AD, since it acts on both pathological hallmarks of AD, tau-hyperphosphorylation [22] and APP cleavage by BACE1.

Methods

Stably transfected SH-SY5Y-APP^{swe} cell line

To generate the stably transfected SH-SY5Y-APP^{swe} neuroblastoma cell-line, SH-SY5Y cells were transfected with pcDNA-3-hAPP695 harboring the Swedish mutation (hAPP695^{swe}) using Effectene (Qiagen) according to the manufacturer's instructions. Selection of successfully transfected cells was started after 72 h using DMEM with 1 mg/ml G418 (Sigma). After 1 week of selection single clones were picked and transferred into 96-well plates (Greiner-bio-one) using DMEM with 1 mg/ml G418. Clones were expanded by cultivation using DMEM with 0.5 mg/ml G418. APP overexpression was tested by western blot.

Primary neurons

Primary cortical neurons were isolated from wild-type mouse embryos at day 14.5. Cortices were collected and cells were dissociated by incubation with Trypsin/EDTA for 6.5 min at 37°C. Cells were cultured in neurobasal medium containing B27 (Invitrogen). Cells were seeded onto plates coated with 0.2 mg/ml poly-D-lysin (Sigma) and 2 μg/ml laminin (Sigma) and incubated at 37°C with 5% CO_2. After 4 days in culture, a complete change of medium was performed and neurons were treated with the respective substances 24 h later.

Treatments

Cells were incubated with the indicated substances as follows: Metformin (MP Biomedicals) at final concentrations up to 2.5 mM for 1 to >24 hours, mTOR inhibitor Temsirolimus (Sigma) at a final concentration of 1 μM for 24 hours, AMPK inhibitor compound C (Calbiochem) at a final concentration of 2.5 μM for 24 hours, S6K1 inhibitor II, DG2 (Calbiochem) at a final concentration of 2.5 μM for 24 hours, and PI3K inhibitor XI, HWT (Calbiochem) at a final concentration of 1 μM for 24 hours.

Western blot

Cell pellets or brain samples were homogenized either in RIPA buffer (20 mM Tris-HCl (pH 7.5), 150 mM NaCl, 1 mM EDTA, 1 mM EGTA, 1% NP-40, 1% sodium deoxycholate, 2.5 mM sodium pyrophosphate, 1 mM β-glycerophosphate, 1 mM Na_3VO_4, 1 μg/ml leupeptin) or in SDS PAGE buffer B (40 mM Tris-HCl pH 6.8, 4% glycerol, 2% SDS, 0.01% bromphenolblue, 2 mM mercaptoethanol), sonicated and boiled for 5 min at 95°C. Proteins were analyzed on 10 or 12% SDS gels or on 16% Tris-Tricine gels (for CTF) and blotted onto PVDF membranes (Roche). Blots were blocked in milk and incubated with the antibodies listed below.

The resulting bands were quantified using AIDA software (Raytest). Statistical analyses were performed using one-way ANOVA with post hoc Dunnett's test to accommodate for multiple comparisons or Student's T-test for two-group comparisons, as appropriate.

Antibodies

BACE1 and TACE antibodies were purchased from Millipore or from Abcam. The following antibodies were purchased from

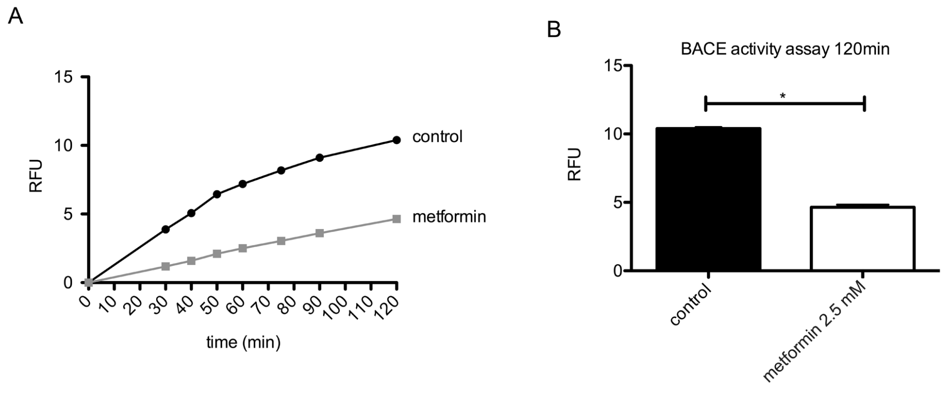

Figure 1. Metformin treatment reduces BACE activity. Primary neurons were treated with or without 2.5 mM metformin for 24 hours. BACE activity in the lysate was analyzed in a fluorometric β-secretase activity assay. (A) The reaction was followed over a time-course of 120 min. Lines represent mean values of fluorescent signal (relative fluorescence units (RFU)). (B) Data at time point 120 min of the assay from (A) is shown in a graph. Columns represent mean values +/− SEM. n = 3 per group, $p < 0.05$.
doi:10.1371/journal.pone.0102420.g001

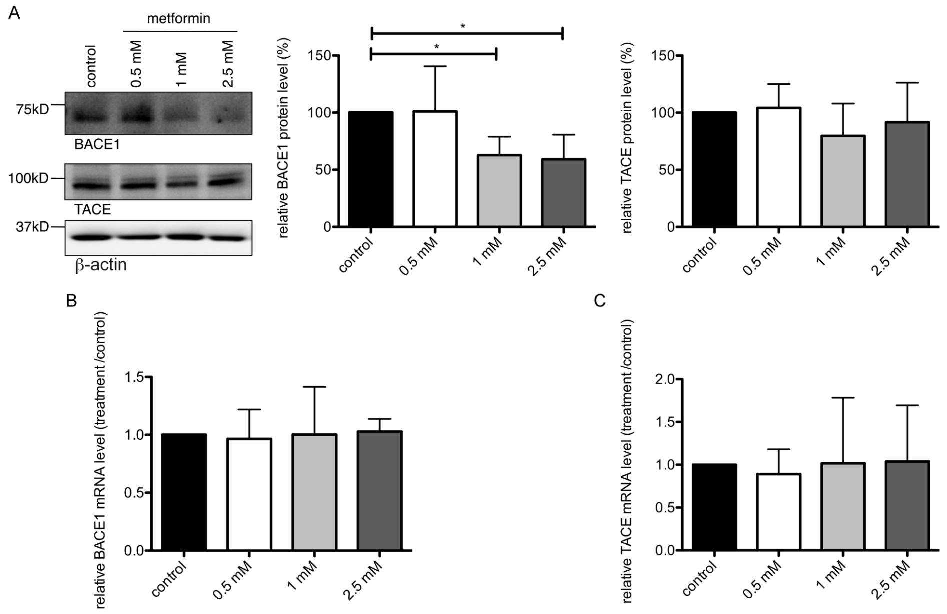

Figure 2. Metformin decreases BACE1 protein level. (A) Primary neurons were treated with increasing concentrations of metformin for 24 hours and BACE1 protein levels were analyzed on western blots using BACE1-, TACE- or β-actin-specific antibodies. Graphs show quantification of western blots, mean values +/− SEM. n = 7 per group, * = $p<0.05$. (B, C) Relative BACE1 and TACE mRNA expression was measured in cells treated as in (A) by means of real-time PCR. Columns represent mean values +/− SEM. n = 8.
doi:10.1371/journal.pone.0102420.g002

Cell signalling: S6, pS6, β-actin and GAPDH. CTF antibodies were purchased from Sigma.

BACE activity assay

BACE activity was measured using the fluorometric β-secretase activity assay (Abcam). Briefly, primary cortical neurons (untreated controls or treated with 2.5 mM metformin for 24 hours) were lysed and the reaction buffer and β-secretase substrate were added. The reaction was measured in a fluorescence plate reader with Ex 335–355 nm and Em 495–510 nm. The β-secretase substrate is a peptide conjugated to the two reporter molecules EDANS and DABCYL. In the un-cleaved form, the fluorescent emissions from EDANS are quenched by the DABCYL moiety. Cleavage of the peptide by the β-secretase separates EDANS and DABCYL leading to a fluorescent signal. β-secretase activity can be expressed as the relative fluorescence units (RFU) per μg of protein sample.

Real-time PCR

Total RNA was isolated using the RNeasy Mini Kit (Qiagen). cDNA was synthesized using the TaqMan reverse transcription reagents kit (Applied Biosystems) and real-time PCR was carried out using the SYBRGreen PCR master mix (Applied Biosystems). Primers used are listed in Table S1.

In vivo treatments

All animal work has been conducted according to relevant national guidelines and has been approved by the responsible authority (Landesamt für Natur, Umwelt und Verbraucherschutz, Nordrhein-Westfalen, permit number 87-51.04.2011. A049/01). Female wild type C57BL/6J mice (age 12 weeks) were treated for 2 weeks with 5 g/l metformin (≈0.03 M) in the drinking water with daily change of water and addition of fresh metformin. Water intake and body weight of the animals were monitored. After 2

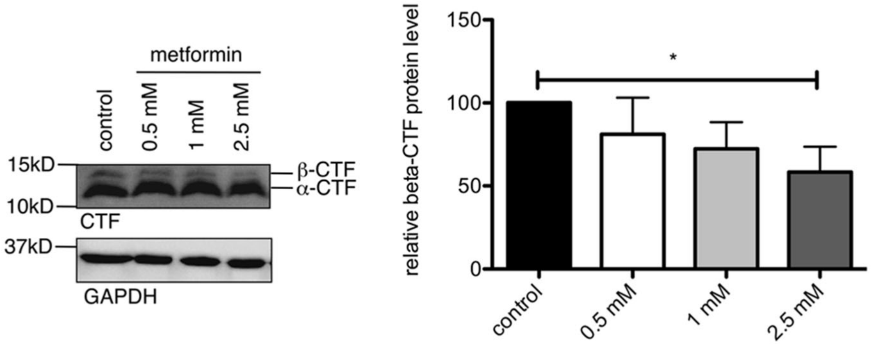

Figure 3. BACE1 cleavage products are reduced after metformin treatment. SH-SY5Y-APP^{swe} cells were treated with increasing concentrations of metformin for 24 hours and protein levels were analyzed on western blots using CTF-specific antibodies. Graphs show quantification of western blots, mean values +/− SEM. n = 4 per group, * = $p<0.05$.
doi:10.1371/journal.pone.0102420.g003

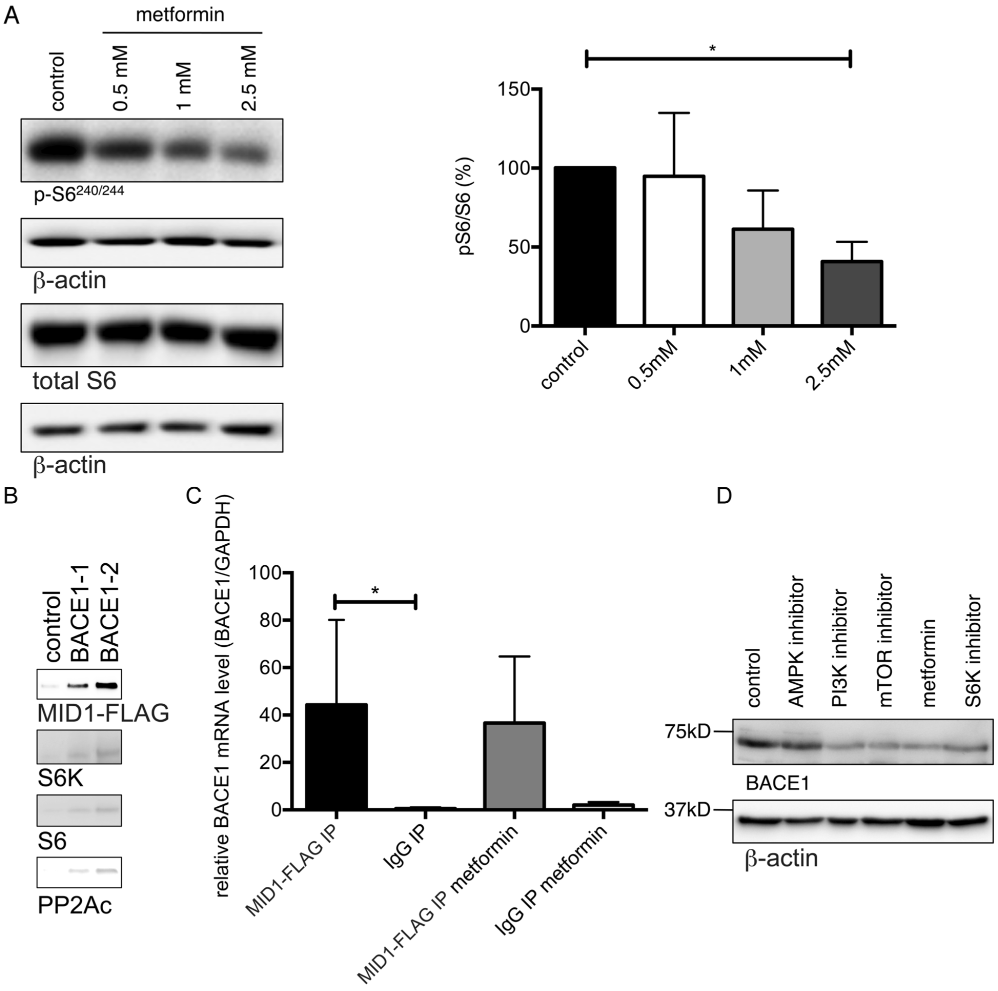

Figure 4. BACE1 translation is regulated by pS6 and inhibited by metformin. (A) Metformin decreases phosphorylation of S6. Primary neurons were treated with increasing concentrations of metformin for 24 hours and phosphorylation of S6 was analyzed on western blots using pS6-, total S6- or β-actin-specific antibodies. Graphs show quantification of western blots, mean values +/− SEM. n = 5 per group, * = $p<0.05$. (B) Two different *in vitro* transcribed, biotinylated BACE1-mRNA fragments containing a putative MIDAS structural motif were incubated with cell lysates that were transfected with MID1-FLAG. RNAs were immobilized on streptavidin-coated magnetic beads. After extensive washing RNA-bound proteins were analyzed by western blotting using antibodies detecting MID1-FLAG, S6K, and S6. As a negative control an experiment with RNAse treatment was performed. (C) RNA immunoprecipitation. Primary neurons were transfected with MID1-FLAG and incubated with or without metformin. Afterwards MID1-mRNPs were purified by immunoprecipitation and MID1-bound mRNAs were analyzed for the presence of BACE1 mRNA using realtime-PCR. Columns represent mean values +/− SEM. n = 4 (D) Kinase inhibitors involved in regulating S6 phosphorylation decrease BACE1 protein levels to a similar extent as metformin. Primary neurons were treated with 2.5 mM metformin, or 1 μM Temsirolimus (an mTOR inhibitor), or 2.5 μM compound C (an AMPK inhibitor), or 2.5 μM DG2 (an S6K1 inhibitor), or 1 μM HWT (a PI3K inhibitor) 24 hours, after which BACE1 protein levels were analyzed on western blots using BACE1- or β-actin-specific antibodies.
doi:10.1371/journal.pone.0102420.g004

weeks of treatment, animals were sacrificed and brains were snap-frozen in liquid nitrogen and broken up using a mortar.

RNA protein pull-down

BACE1 mRNA fragments containing MIDAS-structure-motifs were amplified by PCR, treated with proteinase K and purified by phenol-chloroform extraction. *In vitro* transcription was performed using the RiboMAX Large scale RNA production system-T7 (Promega) in combination with biotinylted UTP. The resulting biotinylated RNAs were purified by phenol-chloroform extraction. After preincubation of RNA with HeLa-protein extracts, RNA pull-down was performed using streptavidin-coated magnetic beads (life technologies).

RNA immunorecipitation

Murine primary neurons were transfected with FLAG-tagged MID1 using $CaCl_2$. 24 hours after transfection cells were treated with or without 2.5 mM metformin and incubated for another 24 h. After UV-crosslinking (200 mJ/cm^2) cells were lysed in TKM buffer (20 mM Tris pH 7.4, 100 mM KCl, 5 mM $MgCl_2$, complete protease inhibitor cocktail (Roche), RNAse inhibitor, 0.2% NP40) and MID1-protein complexes were purified by immunoprecipitation using anti-FLAG-agarose (Sigma) or IgG-agarose (Sigma) as negative control. Protein-bound mRNA was isolated after DNAse and proteinase K digestion by phenol-chloroform purification. Afterwards protein-bound mRNAs were analyzed by realtime-PCR.

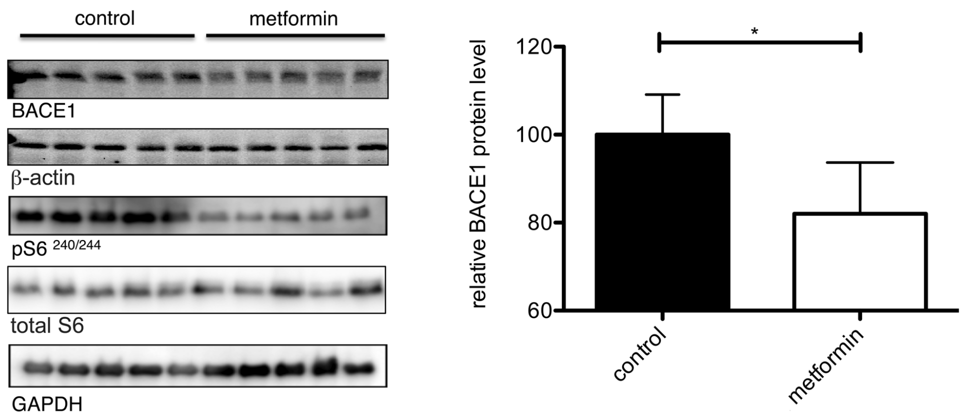

Figure 5. Metformin reduces BACE1 protein levels *in vivo*. Wild type mice were treated for 2 weeks with 5 g/l metformin in the drinking water (≈0.03 M). Brain lysates of these mice were analyzed on western blots detecting BACE1, pS6, total S6, GAPDH and β-actin. Bars represent mean values +/− SEM, (n = 5 per group, *p<0.05).
doi:10.1371/journal.pone.0102420.g005

Results

BACE1 protein levels decrease upon treatment with metformin

In an initial set of experiments we analyzed how metformin affects the β-secretase BACE1 in murine primary neurons. For that, primary neurons of wild-type mice were incubated with 2.5 mM metformin for 24 hours and the activity of the β-secretase BACE1 was analyzed in an enzyme activity assay. Metformin treatment significantly reduced the BACE1 activity compared to untreated control samples (Fig. 1A and 1B). This could be due to either decreased BACE1 protein levels or by reduced BACE1 enzyme activity. To determine which of these is the case, we treated primary neurons with increasing concentrations of metformin for 24 hours and quantified BACE1 protein levels on western blots. We observed a significant reduction of BACE1 protein after metformin treatment (Fig. 2A upper panel). Interestingly, BACE1 mRNA levels did not change after metformin treatment as measured by real-time PCR (Fig. 2B), indicating that BACE1 was not down-regulated at the transcriptional level. Of note, neither protein nor mRNA levels of the α-secretase TACE changed significantly after similar metformin treatment (Fig. 2A, 2C), showing that there was no global translational alteration due to metformin treatment. Together, these data show that metformin treatment decreases the protein expression of BACE1.

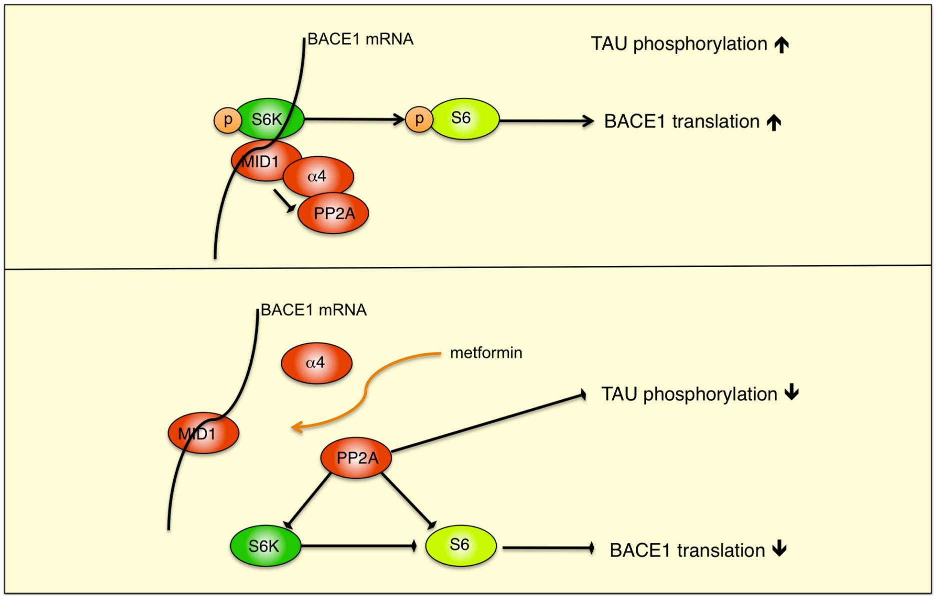

Figure 6. Hypothetical model of the MID1 regulatory complex. A schematic representation of the MID1-PP2A complex with S6K and S6 is shown. MID1 binds to PP2A via its4 regulatory subunit, causing degradation of PP2A and increased phosphorylation of TAU. The MID1 complex also binds to the BACE1 mRNA. By reducing the activity of PP2A and thereby promoting phosphorylation, S6K gets activated and phosphorylates S6. Activated pS6 can then stimulate S6-dependent translation. After metformin treatment, the MID1-complex disassembles and, due to increased PP2A activity, S6-dependent translation of the BACE1 mRNA is reduced and phosphorylation of TAU decreases.
doi:10.1371/journal.pone.0102420.g006

BACE1 cleavage products decrease after treatment with metformin

The first step in Aβ formation is cleavage of full-length APP by the β-secretase BACE1, generating the N-terminal soluble APPβ and the beta C-terminal fragments (β-CTF), which subsequently gives rise to Aβ. In the next set of experiments we investigated these BACE1 cleavage products after metformin treatment in a human cell line model overexpressing the Swedish mutation of APP (SH-SY5Y-APP^{swe}). α- and β-secretase compete for APP as their substrate, with cleavage by the α-secretase being normally favored in cell lines. To combat this, we used the Swedish mutation to promote the amyloidogenic pathway by β-secretase cleavage and increase β-CTF production [26]. This enabled us to detect this cleavage product and the impact of metformin on a direct BACE1 product in cell culture. Metformin treatment resulted in a clear reduction of β-CTF and a decline in BACE1 protein levels (Fig. 3, Fig. S1).

BACE1 translation is regulated by pS6 and inhibited by metformin

The observation that BACE1 protein, but not mRNA, levels are decreased by metformin suggests that metformin may act by inhibiting BACE1 protein translation. As we have shown previously, metformin induces PP2A activity and thereby dephosphorylates PP2A target proteins [22], including the translational regulators p70 S6 kinase (S6K) and ribosomal S6 protein. When phospho-activated, S6K phosphorylates S6, facilitating recruitment and translation of mRNAs containing a 5′ terminal oligopyrimidine tract (5′-TOP) [27]. *In silico* prediction of 5′-TOP motifs in the BACE1 mRNA revealed a typical 5′TOP motif at the transcription start site (CTTTCCG). Therefore, S6K and S6 are good candidates for being translational regulators mediating the metformin effect on BACE1 protein expression. Furthermore, the MID1-PP2A complex binds to and regulates the translation of GC-rich mRNAs. This regulation is achieved by MID1-mediated recruitment of S6K and S6 to the respective mRNAs [24,25]. We performed an *in silico* prediction of the structure of MID1 associated sequences (MIDAS) [24] and analysed the BACE1 mRNA for such structural motifs. In addition to the predicted 5′TOP sequence, the BACE1 mRNA contains several sites that fold like a MIDAS motif. To test if the MID1 complex together with S6K and S6 could be involved in regulating BACE1 translation, we first tested for phosphorylation of S6 in primary neurons with or without metformin treatment on western blots. In line with our previous observations [28], we observed a significant reduction of pS6 after treatment with metformin (Fig. 4A), which coincided with the reduction of BACE1 protein (see Fig. 2). To further validate the connection between the MID1-complex, the phosphorylation/activity of the ribosomal protein S6 and the BACE1 protein levels, we performed an mRNA protein pulldown. *In vitro* transcribed fragments (see Fig. S2) of the BACE1 mRNA containing the predicted MIDAS structural motifs were coupled to beads and incubated with protein extracts of MID1-FLAG overexpressing cells either in the presence or absence of 5 mM metformin. After extensive washing mRNA-bound proteins were analysed on western blots for the presence of MID1-FLAG, S6K and S6. In line with the idea that the MID1-complex together with phospho-activated S6 can regulate BACE1 protein levels, binding of MID1, S6K, S6, and PP2Ac to the BACE1 mRNA probes was detected (Fig. 4B). We confirmed binding of the MID1 protein to endogenous BACE1 mRNA in an RNA immunoprecipitation-assay, in which we analyzed immunopurified MID1-complexes by realtime PCR for MID1-bound mRNAs. Of note, binding of the MID1-protein to the BACE1 mRNA was not changed after treatment of the cells with metformin, suggesting that metformin affects the translation of BACE1 mRNA by dephosphorylating S6K and S6, rather than by inhibiting the mRNA-protein binding between MID1 and the BACE1 mRNA (Fig. 4C). To further validate the connection between phosphorylation/activity of the ribosomal protein S6 and the BACE1 protein levels, we applied a panel of kinase inhibitors, which inhibit diverse kinases involved in insulin signalling. In line with the idea that phospho-activated S6 could regulate BACE1 protein levels, all kinase inhibitors involved in the regulation of S6 phosphorylation decreased BACE1 protein levels to a similar degree as metformin, giving further credence to the idea that phospho-activated S6 can regulate BACE1 levels (Fig. 4D).

BACE1 protein levels decrease upon treatment with metformin *in vivo*

Finally, we wanted to test metformin's effects on BACE1 expression in an *in vivo* setting. To this end, we treated wild-type C57BL/6J mice for 2 weeks with 5 g/l metformin (≈0.03 M) in the drinking water. Afterwards, whole brain lysates were analyzed on western blots. In agreement with our cell culture findings, metformin treated animals had significantly decreased BACE1 protein levels compared to untreated controls. Additionally, S6 phosphorylation also decreased in the treated animals (Fig. 5). Of note, neither the daily water intake nor the body weight of treated animals differed significantly from non-treated control animals (Fig. S3). Together, our data indicate that metformin treatment can decrease BACE1 protein expression and activity, thereby reducing BACE1 cleavage products and Aβ production.

Discussion

Alzheimer's disease (AD) is characterized by two pathological hallmarks: senile plaques, which are composed of Aβ peptides, and neurofibrillary tangles, which are composed of hyperphosphorylated TAU protein. In this study, we investigated the effect of metformin on the main amyloidogenic enzyme BACE1 and its direct cleavage product β-CTF, the precursor substrate for the production of Aβ peptides. We show that treatment with the anti-diabetic drug metformin decreases BACE1 protein expression (see model Fig. 6) and activity, thereby reducing BACE1 cleavage products.

Metformin treatment of a cell model for insulin resistance has previously been reported to attenuate Aβ production, in agreement with our observation of metformin inhibiting BACE1 protein production [29]. There is not universal support of this finding in the literature, with one report of metformin treated primary neurons showing increased BACE1 levels [30]. These different results may be due to differences in used metformin concentrations or other protocol/culture differences. While Chen et al. observed increased BACE1 mRNA levels, protein levels and Aβ production in cell cultures in the absence of insulin, addition of insulin to the culture medium reduced Aβ production. This reduction could even be enhanced by addition of metformin [30]. This reduction is in agreement with our own observations, since we have performed all of our experiments in the presence of insulin.

Under normal conditions, BACE1 cleaves Aβ at Glu11. Further cleavage by γ-secretase leads to a truncated non-amyloidogenic Aβ isoform. Introducing the Swedish mutation into APP results in a switch of the cleavage site, and thus cleavage at Asp1 generating β-CTF. Cleavage of β-CTF by γ-secretase produces amyloidogenic Aβ. Therefore, cleavage at the Asp1 is enhanced in AD models carrying the Swedish mutation [2]. Metformin, by reducing

BACE1 levels, most likely reduces cleavage at both Glu11 and Asp1, since we have detected reduction of β-CTF in a cell line model carrying the Swedish mutation as well as reduced β-secretase activity in wildtype neurons.

In addition, we show that metformin reduces BACE1 protein levels in wild type mice (C57BL/6J). Interestingly, Wang et al. observed in another non-AD mouse model (C57/129J) that metformin enhances spatial memory formation, probably by promoting neurogenesis [31]. In obese, leptin-resistant mice, metformin attenuates AD-like phospho-tau neuropathology. Furthermore, Aβ levels in these mice are increased and this increase is attenuated by both metformin and saline treatment [32]. In contrast to the settings in our study, these mice were diabetic and injected with metformin for 18 weeks. Clearly, future studies in different AD mouse models are needed to investigate the effect of metformin on the AD pathology more deeply.

Translation of several proteins implicated in neurodegeneration is regulated by complexes containing the ubiquitin ligase MID1 [24,25,33]. This regulation is achieved by MID1-mediated recruitment of the translational regulators S6K and S6 to mRNA [24,25]. When phospho-activated, S6K phosphorylates S6, facilitating recruitment and translation of associated mRNAs [27]. Here, we show that BACE1 mRNA binds to MID1, suggesting that BACE1 mRNA translation is also initiated/regulated by the MID1 regulatory complex. Since metformin interferes with the assembly of the MID1 protein complex, it thereby reduces translation of BACE1 mRNA (see model Fig. 5), leading to decreased β-secretase activity in the cell. As BACE1 is responsible for formation of cytotoxic Aβ peptides, reduced β-secretase activity may result in diminished Aβ production. Additionally, metformin also decreases tau-phosphorylation by disassembly of the MID1 protein complex, leading to reduced PP2A activity [22]. The targeting of both major pathological hallmarks of AD makes metformin a particularly interesting candidate for treating the AD neuropathology.

The MID1 protein complex not only regulates PP2A activity but also the activity of the PP2A opposing kinase mTOR. Though it has an inhibitory function on PP2A [23], MID1 positively regulates mTOR [34]. Among other effects, metformin disassembles the MID1 complex [22], thereby activating PP2A and suppressing mTOR. Decreased mTOR signalling results in (i) activation of autophagy and (ii) decreased translation of mRNAs regulated by its downstream effectors S6K and 4E-BP1 [35]. However, metformin's effect on MID1/PP2A and mTOR-dependent translation will most likely not be specific for BACE1, with translation of other mRNAs that are regulated by PP2A/mTOR also likely reduced. Both induction of autophagy and reduced protein translation are especially vital for the adult nervous system, since these processes alter Aβ and phospho-TAU homeostasis toward less toxic protein species [17,36]. Furthermore, metformin is widely used as an anti-diabetic medication and is generally well tolerated. Even if translation of multiple mRNAs regulated by S6K is suppressed by metformin, this does not seem to be harmful. Therefore, targeting mTOR/PP2A with metformin seems to represent a reasonable therapeutic approach to interfering in the development/progression of AD. Future *in vivo* studies in models of AD are needed to strengthen this hypothesis.

Supporting Information

Figure S1 Metformin decreases BACE1 protein level. SH-SY5Y-APPswe cells were treated with increasing concentrations of metformin for 24 h and protein levels were analyzed on western blots using BACE1-, TACE- or β-actin-specific antibodies.
(TIF)

Figure S2 Schematic representation of the BACE1 mRNA transcript. The translational start (ATG) and stop codons (TGA) are indicated. Furthermore the predicted 5′TOP and two of the predicted MIDAS motifs are illustrated. Primers for in vitro amplification and in vitro transcription for mRNA pull-down assays are indicated as arrows.
(TIF)

Figure S3 Metformin does not influence body weight or water intake. Wild type mice were treated for 2 weeks with metformin in the drinking water. Body weight and water intake of these experimental animals are shown. Columns represent mean values +/− SEM, (n = 5 per group).
(TIF)

Table S1 Primer sequences. Sequences of primers used in this study are listed (in 5′→3′ direction).
(DOCX)

Acknowledgments

We thank Prof. Dieter Edbauer for kindly providing the APP^{swe} construct. We thank Gabor Petzold (DZNE) and Erdem Tamgüney (DZNE) for critical reading of the paper.

Author Contributions

Conceived and designed the experiments: MH DE SK. Performed the experiments: MH FM NG SS SD SK. Analyzed the data: MH FM DR SK. Contributed reagents/materials/analysis tools: DR. Wrote the paper: SK.

References

1. Alzheimer A, Stelzmann RA, Schnitzlein HN, Murtagh FR (1995) An English translation of Alzheimer's 1907 paper, "Uber eine eigenartige Erkankung der Hirnrinde". Clin Anat 8: 429–431.
2. Deng Y, Wang Z, Wang R, Zhang X, Zhang S, et al. (2013) Amyloid-beta protein (Abeta) Glu11 is the major beta-secretase site of beta-site amyloid-beta precursor protein-cleaving enzyme 1(BACE1), and shifting the cleavage site to Abeta Asp1 contributes to Alzheimer pathogenesis. Eur J Neurosci 37: 1962–1969.
3. Zhang YW, Thompson R, Zhang H, Xu H (2011) APP processing in Alzheimer's disease. Mol Brain 4: 3.
4. Shankar GM, Walsh DM (2009) Alzheimer's disease: synaptic dysfunction and Abeta. Mol Neurodegener 4: 48.
5. Bennett BD, Babu-Khan S, Loeloff R, Louis JC, Curran E, et al. (2000) Expression analysis of BACE2 in brain and peripheral tissues. J Biol Chem 275: 20647–20651.
6. Southan C, Hancock JM (2013) A tale of two drug targets: the evolutionary history of BACE1 and BACE2. Front Genet 4: 293.
7. Sun X, He G, Song W (2006) BACE2, as a novel APP theta-secretase, is not responsible for the pathogenesis of Alzheimer's disease in Down syndrome. FASEB J 20: 1369–1376.
8. Alonso AD, Grundke-Iqbal I, Barra HS, Iqbal K (1997) Abnormal phosphorylation of tau and the mechanism of Alzheimer neurofibrillary degeneration: sequestration of microtubule-associated proteins 1 and 2 and the disassembly of microtubules by the abnormal tau. Proc Natl Acad Sci USA 94: 298–303.
9. Gong CX, Liu F, Grundke-Iqbal I, Iqbal K (2005) Post-translational modifications of tau protein in Alzheimer's disease. J Neural Transm 112: 813–838.
10. Tian Q, Wang J (2002) Role of serine/threonine protein phosphatase in Alzheimer's disease. Neurosignals 11: 262–269.
11. Brands AM, Biessels GJ, de Haan EH, Kappelle LJ, Kessels RP (2005) The effects of type 1 diabetes on cognitive performance: a meta-analysis. Diabetes Care 28: 726–735.
12. Strachan MW, Deary IJ, Ewing FM, Frier BM (1997) Is type II diabetes associated with an increased risk of cognitive dysfunction? A critical review of published studies. Diabetes Care 20: 438–445.

13. Ott A, Stolk RP, van Harskamp F, Pols HA, Hofman A, et al. (1999) Diabetes mellitus and the risk of dementia: The Rotterdam Study. Neurology 53: 1937–1942.
14. Bertrand L, Horman S, Beauloye C, Vanoverschelde JL (2008) Insulin signalling in the heart. Cardiovasc Res 79: 238–248.
15. Ravikumar B, Sarkar S, Davies JE, Futter M, Garcia-Arencibia M, et al. (2010) Regulation of mammalian autophagy in physiology and pathophysiology. Physiol Rev 90: 1383–1435.
16. Fritsche L, Weigert C, Haring HU, Lehmann R (2008) How insulin receptor substrate proteins regulate the metabolic capacity of the liver–implications for health and disease. Curr Med Chem 15: 1316–1329.
17. O'Neill C, Kiely AP, Coakley MF, Manning S, Long-Smith CM (2012) Insulin and IGF-1 signalling: longevity, protein homoeostasis and Alzheimer's disease. Biochem Soc Trans 40: 721–727.
18. Jolivalt CG, Lee CA, Beiswenger KK, Smith JL, Orlov M, et al. (2008) Defective insulin signaling pathway and increased glycogen synthase kinase-3 activity in the brain of diabetic mice: parallels with Alzheimer's disease and correction by insulin. J Neurosci Res 86: 3265–3274.
19. Li ZG, Zhang W, Sima AA (2007) Alzheimer-like changes in rat models of spontaneous diabetes. Diabetes 56: 1817–1824.
20. Kirpichnikov D, McFarlane SI, Sowers JR (2002) Metformin: an update. Ann Intern Med 137: 25–33.
21. Knowler WC, Barrett-Connor E, Fowler SE, Hamman RF, Lachin JM, et al. (2002) Reduction in the incidence of type 2 diabetes with lifestyle intervention or metformin. N Engl J Med 346: 393–403.
22. Kickstein E, Krauss S, Thornhill P, Rutschow D, Zeller R, et al. (2010) Biguanide metformin acts on tau phosphorylation via mTOR/protein phosphatase 2A (PP2A) signaling. Proc Natl Acad Sci USA.
23. Trockenbacher A, Suckow V, Foerster J, Winter J, Krauss S, et al. (2001) MID1, mutated in Opitz syndrome, encodes an ubiquitin ligase that targets phosphatase 2A for degradation. Nat Genet 29: 287–294.
24. Aranda-Orgilles B, Rutschow D, Zeller R, Karagiannidis AI, Kohler A, et al. (2011) Protein phosphatase 2A (PP2A)-specific ubiquitin ligase MID1 is a sequence-dependent regulator of translation efficiency controlling 3-phosphoinositide-dependent protein kinase-1 (PDPK-1). J Biol Chem 286: 39945–39957.
25. Krauss S, Griesche N, Jastrzebska E, Chen C, Rutschow D, et al. (2013) Translation of HTT mRNA with expanded CAG repeats is regulated by the MID1-PP2A protein complex. Nat Commun 4: 1511.
26. Citron M, Oltersdorf T, Haass C, McConlogue L, Hung AY, et al. (1992) Mutation of the beta-amyloid precursor protein in familial Alzheimer's disease increases beta-protein production. Nature 360: 672–674.
27. Dufner A, Thomas G (1999) Ribosomal S6 kinase signaling and the control of translation. Exp Cell Res 253: 100–109.
28. Kickstein E, Krauss S, Thornhill P, Rutschow D, Zeller R, et al. (2010) Biguanide metformin acts on tau phosphorylation via mTOR/protein phosphatase 2A (PP2A) signaling. Proc Natl Acad Sci USA 107: 21830–21835.
29. Gupta A, Bisht B, Dey CS (2011) Peripheral insulin-sensitizer drug metformin ameliorates neuronal insulin resistance and Alzheimer's-like changes. Neuropharmacology 60: 910–920.
30. Chen Y, Zhou K, Wang R, Liu Y, Kwak YD, et al. (2009) Antidiabetic drug metformin (GlucophageR) increases biogenesis of Alzheimer's amyloid peptides via up-regulating BACE1 transcription. Proc Natl Acad Sci USA 106: 3907–3912.
31. Wang J, Gallagher D, DeVito LM, Cancino GI, Tsui D, et al. (2012) Metformin activates an atypical PKC-CBP pathway to promote neurogenesis and enhance spatial memory formation. Cell Stem Cell 11: 23–35.
32. Li J, Deng J, Sheng W, Zuo Z (2012) Metformin attenuates Alzheimer's disease-like neuropathology in obese, leptin-resistant mice. Pharmacol Biochem Behav 101: 564–574.
33. Aranda-Orgilles B, Trockenbacher A, Winter J, Aigner J, Kohler A, et al. (2008) The Opitz syndrome gene product MID1 assembles a microtubule-associated ribonucleoprotein complex. Hum Genet 123: 163–176.
34. Liu E, Knutzen CA, Krauss S, Schweiger S, Chiang GG (2011) Control of mTORC1 signaling by the Opitz syndrome protein MID1. Proc Natl Acad Sci USA 108: 8680–8685.
35. Wullschleger S, Loewith R, Hall MN (2006) TOR signaling in growth and metabolism. Cell 124: 471–484.
36. Caccamo A, Majumder S, Richardson A, Strong R, Oddo S (2010) Molecular interplay between mammalian target of rapamycin (mTOR), amyloid-beta, and Tau: effects on cognitive impairments. J Biol Chem 285: 13107–13120.
